# Organising Graduate Instruction

**Published:** 1910-04-16

**Authors:** 


### SPECIAL ARTICLE.

### ORGANISING GRADUATE INSTRUCTION.

### THE INTERNATIONAL COMMITTEE FOR MEDICAL EXTENSION COURSES.

Last week we published an article by Professor Kutner, C.V.O., drawing attention to the urgent need for systematisation in graduate instruction in medicine, and giving a *résumé* of what has already been done and what has yet to be achieved before such study can be said to be definitely organised on a basis of mutual co-operation. The article embodied the substance of Professor Kutner's remarks delivered at the sectional meeting of the Budapest Conference last year—barely six months ago—when it was decided, by representatives of practically all the countries alluded to in the article, to establish a central international committee for the purpose of definitely dealing with the development of graduate study in medicine. A preliminary series of resolutions was drawn up at the Conference, together with an outline constitution and tentative rules. Since then all these have been revised and scrutinised, the Governments of the various countries have loyally accepted the invitation to send delegates to the central committee, and the organisation which in the future is to control the development of graduate study has at last been properly established. With the creation of the International Committee for Medical Extension Courses graduate study in medicine enters a new era, and graduate students all the world over will be grateful that the first step towards systematisation and organisation, co-operation and mutual aid, has at last been taken, and above all that its prospects of success are so promising.

### What Graduate Instruction in Medicine Should Be.

Once more it is worth while to set forth the ideals which a successful scheme of graduate study in medicine should attain. Medical education is not to be considered finished at the end of the curriculum. The constant progress of science requires on the part of the practitioner an equally constant effort in the direction of further study, and practitioners must be given opportunities to supplement their knowledge without material sacrifice on their part. Therefore, the arrangements which are to serve this purpose—*i.e.* any scheme of graduate instruction—ought to be (1) gratuitous, (2) in the place of domicile of the attending practitioners or close to the same, and (3) held at such times as to be convenient for the practitioner to attend. There should, therefore, be created in the large cities scientific centres, educational institutions in connection with which the local hospitals should, as far as possible, be utilised, and in which practitioners capable of acting as teachers should serve as lecturers. Lectures given here, at such centres should embrace all clinical and theoretical subjects, as well as subjects in the corellated sciences, such as public health, social hygiene, etc. While at the present moment lectures, demonstrations, and practical courses must be accepted as the best form of continued study, it should be the aim in the future to create as many institutions as possible serving exclusively the purpose of medical post-graduate instruction.

These in brief are the main points which should be borne in mind by those interested in the extension of graduate instruction in medicine, and, as will be seen from the statutes and membership of the newly formed committee, there is every prospect that the organisation will keep these ideals steadily in view and work on the lines so carefully laid down. Through the courtesy of the General Secretary we are enabled to give an advance copy of the statutes and list of members.

### STATUTES.

NAME.—Art. 1: The name of the Association is "The International Committee for Medical Extension Courses."

OBJECTS.—Art. 2: The object of the "International Committee for Medical Extension Courses" (graduate courses for practitioners) is to co-operate in all matters pertaining to medical after-college training which may be advanced by such co-operation. These are, among others—Investigations regarding the organisation of medical extension courses in the different countries and reports on the arrangements (especially the institutions) for medical extension courses created for mutual stimulation and instruction; furthermore, investigations concerning the relations between academical instruction and after-college training existing in the different countries; information regarding educational appliances, opportunities for after-college training in the different countries; exchange of eminent scholars for the purpose of instructing physicians on certain important innovations, arrangement of conferences in connection with international medical congresses, statistical work, the promotion of journeys to foreign countries for the purpose of medical study, etc.

MEMBERS COMPOSING THE ASSOCIATION.—Art. 3: The Association consists of ordinary members, extraordinary members, and honorary members.

Art. 4: The ordinary members are elected by the central associations in the different countries charged with the promotion of the medical after-college training. Each country is represented by three members. Countries with more than ten millions of inhabitants get one additional representative for each five millions over and above that number; but the total number of representatives for one country shall not exceed five. A union of States is regarded as one country. In countries where there are no special central associations for the promotion of medical extension courses, delegates to the international association may be nominated by the Government or by medical organisations.

Art. 5: As extraordinary members may be appointed persons having distinguished themselves in their efforts on behalf of the development of medical after-college training and whom it is intended to bring into permanent connection with the International Committee; extraordinary members are not entitled to vote.

Art. 6: As honorary members may be appointed persons who have rendered services of permanent value in the cause of medical after-college training; honorary members have the same rights as ordinary members.

ADMINISTRATION.—Art. 7: The business of the Association is conducted by:—(1) The Board of Management (Art. 8, etc.). (2) The Board of Directors (Arts. 11, 12, 13).

BOARD OF MANAGEMENT.—Art. 8: The administration of the affairs of the Association devolves on the Board of Management. The Committee is composed of a President and five members. The President and the other members are elected by the Board of Directors at its ordinary meeting for the three following business years, and are eligible for re-election. In case of a parity of votes the President's vote decides. The Board of Management has its seat in a capital of the countries participating.

Art. 9 : All the ordinary business that is not transacted by the President himself devolves on the Secretary-General of the Committee, who is elected by the Board of Management for a term of three years.

Art. 10 : The Board of Management is empowered to appoint assistants in the different countries with the consent of delegates from the country in question, to help the Secretary-General in his business.

BOARD OF DIRECTORS.—Art. 11 : The Board of Directors consists of a number of members equal to the number of countries represented in the Committee. The members may in case of their unavoidable absence nominate their own representatives. Honorary members have the right to speak and vote at all meetings.

Art. 12 : The ordinary meetings of the Board of Directors take place in connection with the general meetings. Extraordinary meetings are to be convened when the Board of Management thinks them necessary.

Art. 13 : The Board of Directors decides what measures shall be taken for the furtherance of the aims of the Association, besides this it receives the business report of the Board of Management, decides about the budget prepared by the Board of Management, and gives a discharge to the same for its administrative responsibilities of the preceding year.

GENERAL COUNCIL.—Art. 14 : The Association meets, as a rule, every three years ; at its meeting which shall, whenever possible, coincide with an International Congress, a report shall be made of the work done by the Association. Besides the election of extraordinary and honorary members (Arts. 5, 6), the right to alter the statutes of the Association belongs to it alone. Such alterations can be made only on the proposal of directors and with the approval of a majority of two-thirds of the members present.

FINANCE.—The expenses of the Association are met by dues and donations.

### Membership.

The committee is composed as follows :—

President.—Geh. Med.-Rat Prof. Dr. Waldeyer, (Berlin). Administrative Committee.—The President and the Hon. Secretary, Hofrat Prof. Dr. E. von Grósz (Budapest), Prof. Dr. Landouzy (Paris), Dr. F. W. Pavy, F.R.S. (London), Excellency Prof. Dr. von Rein (St. Petersburg). General Secretary.—Prof. Dr. R. Kutner (Berlin). Members (with power to co-opt).—*Belgium* : President Dr. Casse, Dr. Léopold Dejace, Prof. Dr. Firket (all of Liege) ; *Bulgaria* : Dr. J. Georgieff (Roustschouk), Dr. M. Iwanoff and Dr. D. Kiroff (of Sofia) ; *Denmark* : Prof Dr. Faber, Dr. Rördam, and Prof. Rovsing (of Copenhagen) ; *Germany* : Kgl. Geheimer Rat Prof. Dr. von Angerer (Munich) ; *England* : Dr. Jamieson (Edinburgh), Dr. F. W. Pavy (London), Dr. Pickthorn, R.N., and Sir B. Franklin and Sir R. Havelock-Charles (appointed by the Indian Office) ; *France* : Dr. Lucas-Championnière (Paris), Prof. Dr. Landouzy (Paris), Prof. Dr. Tripier (Lyons), Dr. R. Blondel (Paris), as co-opted member ; *Greece* : Prof. Dr. Kallionzis, Priv.-Doz. Dr. Mermingas, and Prof. Dr. Savas (of Athens) ; *Italy* : Exc. Prof. Dr. G. Baccelli (Rome), Prof. Dr. C. Golgi, Prof. Dr. E. Maragliano (Genoa) ; *Norway* : M. Holmboe (Chef du Musée Médicinal, Christiania), Prof. Dr. Johannessen, Prof. W. Uchermann (Christiania) ; *Austria* : Hofrat Prof. Dr. Frhr. A. von Eiselsberg, Ministerialrat Dr. F. Ritter v. Haberler (Vienna), and Dr. J. Thenen (Vienna) ; *Russia* : Excellency Prof. Dr. von Rein (St. Petersburg) ; *Sweden* : Prof. Dr. J. Hammar (Upsala), Prof. Dr. Petrén (Upsala), Med.-Rat Dr. Wawrinsky (Stockholm) ; *Switzerland* : Prof. Dr. Bourget (Lausanne), Dr. G. Feurer (St. Gall), Prof. Dr. Kocher (Berne) ; *Hungary* : Prof. Dr. E. von Grósz, Prof. Dr. Baron von Korányi, and Prof. Dr. C. Müller (Budapest). Central Office.—Kaiserin Friedrich-Haus, Luisenplatz 2-4, Berlin NW 6.

The representatives are appointed, for the present, by the respective Governments, two of the English members being nominated by the India Office, while the others are appointed by the Foreign Office. The committee has power to co-opt members, and will no doubt exercise that right in order to obtain the help of graduate teachers, deans of graduate schools, and those interested in the movement.

### Work of the Association.

For the present the committee confines itself to gathering information that, being authoritative, official, and reliable, may help it in its future work. After consultation the following scheme of questions has been drawn up for exhaustive consideration, and the committee will be grateful for any information with regard to the various points that may be forwarded to the general secretary.

#### A.—Academic University Instruction.

I. *Instruction* :

1. Details of the preliminary scientific instruction (anatomy, physiology, general natural sciences, in particular chemistry, physics, botany, zoology). 2. Details of instruction in pathological anatomy, bacteriology, and hygiene. 3. Details of instruction in special subjects. 4. Instruction in social medicine. *Note.*—Special value is attached to acquiring knowledge of the organisation of the individual instruction (lectures, instruction at the bedside of the patient, practical exercises, instruction for the students as a whole or in single groups, number of lessons, plans of study, etc.).

II. *Legal regulations for the course of study and examination.*

III. *Organisation of the University instruction for Military Surgeons.*

#### B.—Medical Extension Courses.

I. *Organisation of Medical Extension Courses (including statements in regard to time, place, and the question of fees).*

1. Associations for medical extension courses (statutes). 2. Medical extension courses at the University.

II. *Special Institutes for Medical Extension Courses.*

1. Clinical or policlinical institutes. 2. Academies (and similar teaching institutes).

#### C. Medical Educational Appliances.

I. *Auxiliaries for the intuitive method of instruction* (object lessons).

II. *Institutes for educational appliances, collections, particularly eminent supply houses, etc.*

The first meeting of the general committee will be held at an early date, and the agenda for that meeting includes such subjects as the discussion of the questions formulated and possible alterations, the organisation of collective investigation committees, of an international information bureau for medical graduates, and the discussion on the financial problems raised by the formation of the Association. We hope to give early and definite information regarding the time and place of the meeting as soon as such information is available.

